# Supplementary material for: Evaluating de Novo Assembly and Binning Strategies for Time Series Drinking Water Metagenomes
Source: Microbiol Spectr. 2021 Nov 3;9(3):e01434-21. doi: 10.1128/Spectrum.01434-21 (PMC8567270; doi:10.1128/Spectrum.01434-21)
Supplement: SUPPLEMENTAL FILE 1 — Supplemental material. Download SPECTRUM01434-21_Supp_1_seq3.pdf, PDF file, 0.4 MB [file spectrum01434-21_supp_1_seq3.pdf]

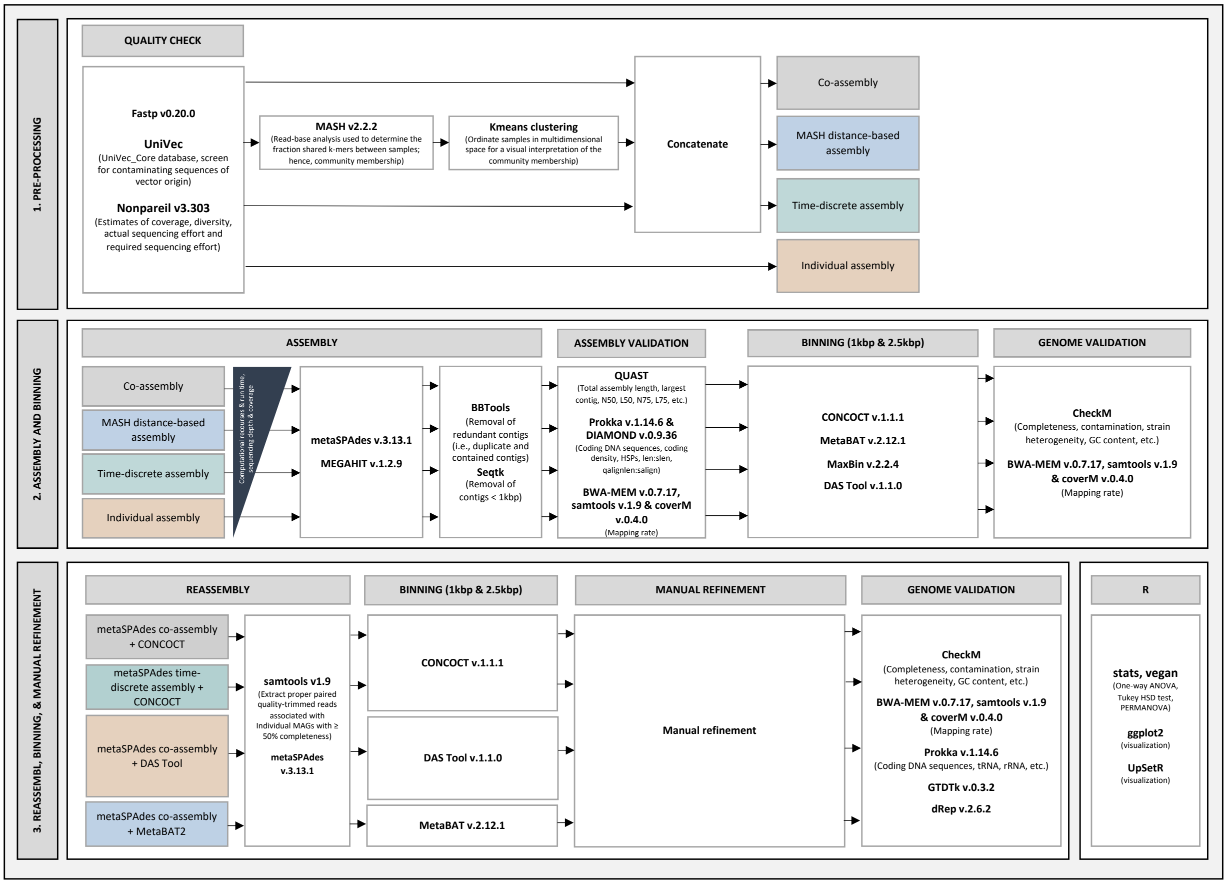

Figure S1 Workflow used for the processing of time-series metagenomic sequencing data. In this pipeline the performance of a combination of assembly (metaSPAdes and MEGAHIT) and binning software (CONCOCT, MetaBAT2, MaxBin2, and DAS Tool) were evaluated using four assembly strategies, including individual assembly and three co-assembly approaches, i.e., co-assembly with all samples, MASH distance-based assembly, and time-discrete assembly. This resulted in 32 combinations of assembler, assembly strategy, and binning approaches (Table S2).

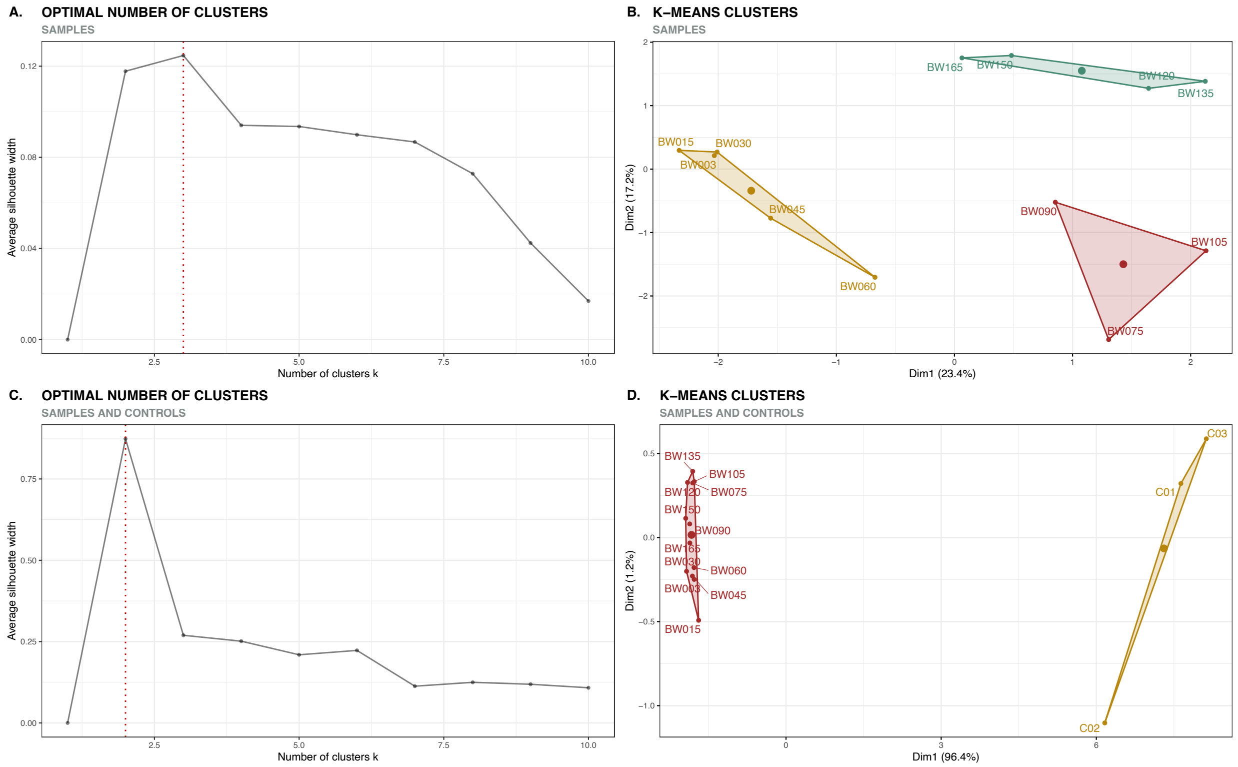

Figure S2 K-means clustering of samples using MASH dissimilarity indices. **A)** Optimal number of clusters for samples as determined using the average silhouette method. **B)** Three distinct clusters were identified amongst samples representing BW003, BW015, BW030, BW045 and BW060 (yellow), BW075, BW090 and BW105 (red) and BW120, BW135, BW150 and BW165 (green). **C)** Optimal number of clusters for samples and controls as determined using the average silhouette method. Three distinct clusters were identified amongst samples representing BW003, BW015, BW030, BW045 and BW060 (yellow), BW075, BW090 and BW105 (red) and BW120, BW135, BW150 and BW165 (green). **D)** Two clusters grouping controls (C01, C02 and C03) independently from samples. Abbreviations: BW, bulk water; C, Controls.

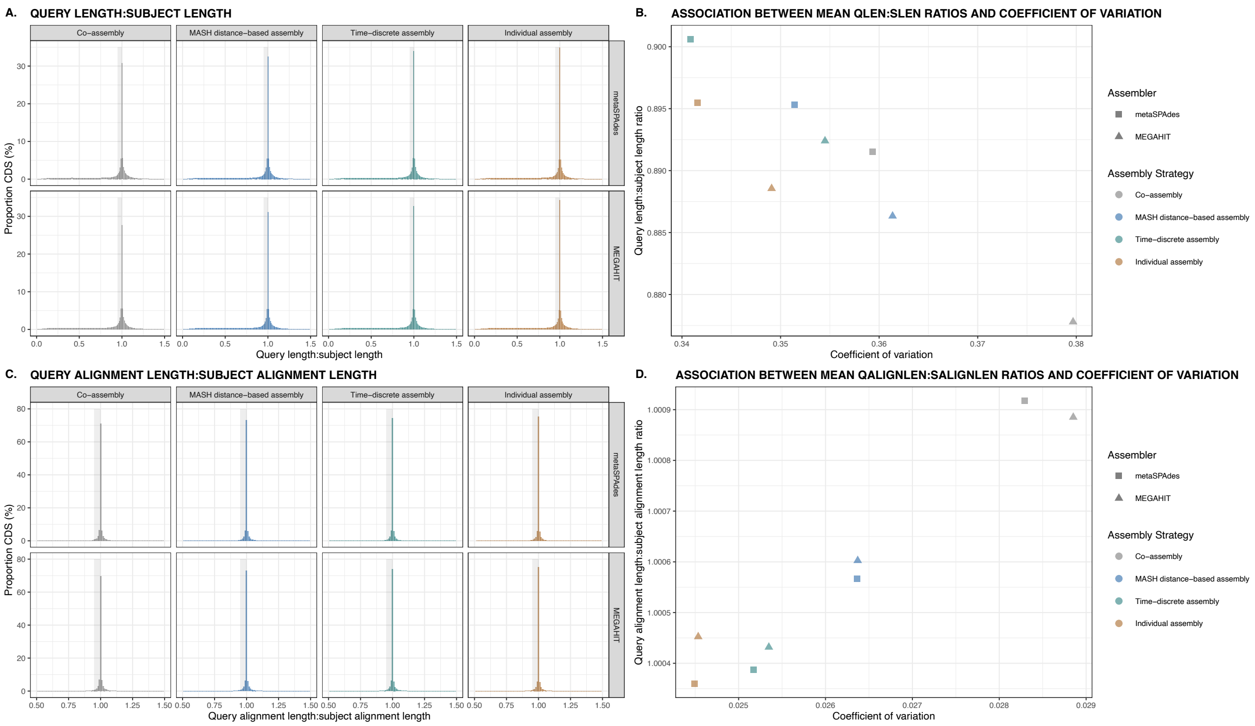

Figure S3 A) Histogram displaying the proportion coding DNA sequences (CDSs) of the metaSPAdes (■) and MEGAHIT (▲) assembly strategies (i.e., co-assembly of al samples (grey), MASH distance-based assembly (blue), time-discrete assembly (green), and individual assembly (orange)) with relation to their query to subject sequence length ratios (qlen/slen). The gray shade indicates qlen:slen ratios ranging between 0.95 and 1.0. **B)** Association between the mean qlen:slen ratios and coefficient of variance (C_v) estimates of the metaSPAdes and MEGAHIT assembly strategies. **C)** Histogram displaying the proportion CDSs of the metaSPAdes and MEGAHIT assembly strategies with relation to their query to subject sequence alignment length ratios (qalignlen/salignlen). The gray shade indicates qalignlen:salignlen ratios ranging between 0.95 and 1.0. **D)** Association between the mean qlen:slen ratios and C_v estimates of the metaSPAdes and MEGAHIT assembly strategies.

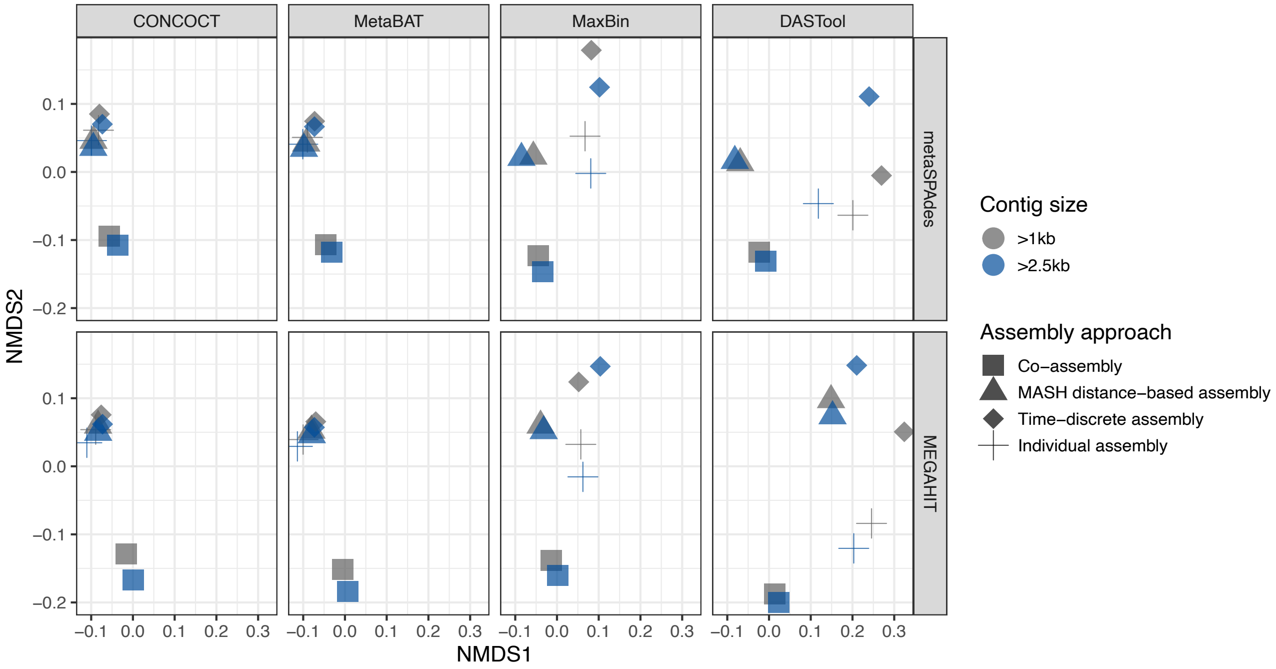

Figure S4 Non-metric multidimensional scaling (NMDS) based on MASH distances of unrefined bins (completeness > 50%) associated with 64 assembly/binning approaches that where reconstructed with assemblies containing contigs greater than 1kbp (grey) or 2.5kbp (blue) using CONCOCT, MetaBAT, MaxBin and DAS Tool. PERMANOVA results in relation to assembly/binning approach and contig size revealed that that the assembly/binning approach explained approximately 96% (PERMANOVA, $F(31) = 46.24$, $R^2 = 0.96$, $p < .05$) of the variation, while contig size explained 2% (PERMANOVA, $F(1) = 36.16$, $R^2 = 0.02$, $p < .05$) .

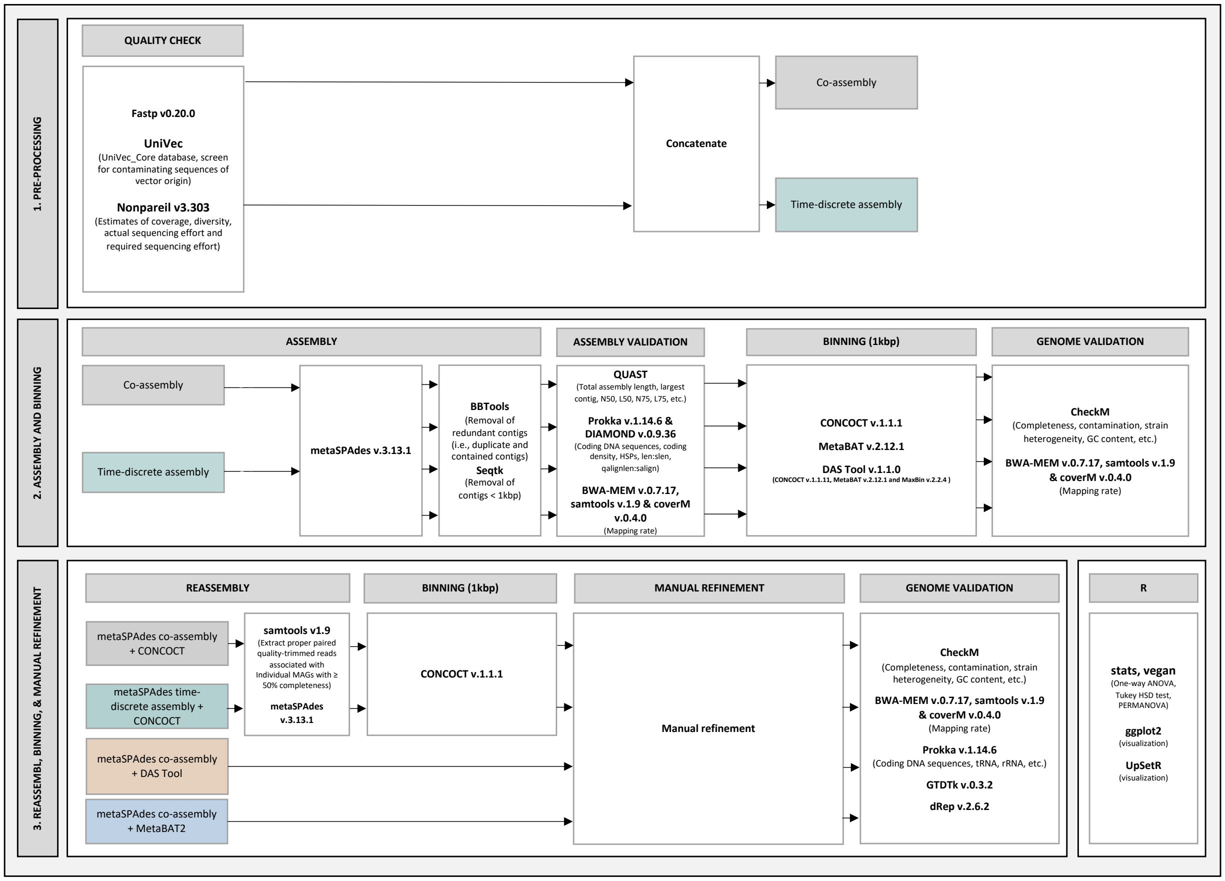

Figure S5 Workflow used for the processing of time-series metagenomic sequencing data resulting in the highest quality and number MAGs. In this pipeline, assembly with metaSPAdes and binning with CONCOCT, MetaBAT2, and DAS Tool follow by dereplication maximize the recovery of non-redundant MAGs.
